# Supplementary material for: Drosophila melanogaster Systemic Infection Model to Study Altered Virulence during Polymicrobial Infection by Aeromonas
Source: Pathogens. 2023 Mar 2;12(3):405. doi: 10.3390/pathogens12030405 (PMC10055632; doi:10.3390/pathogens12030405)
Supplement: Supplementary file 1 [file pathogens-12-00405-s001.zip › pathogens-2123419-supplementary.pdf]

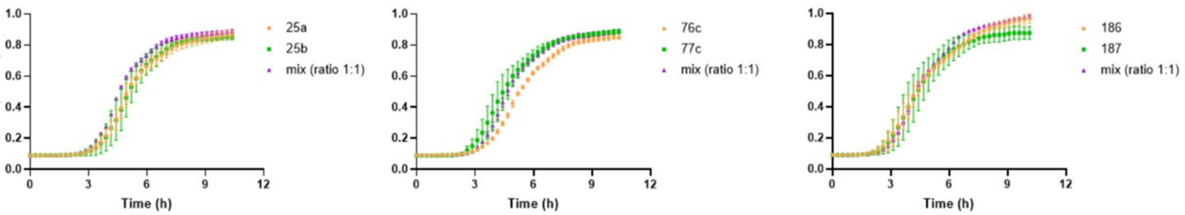

Figure S1: growth curves in LB of single strains and paired strains used in this study

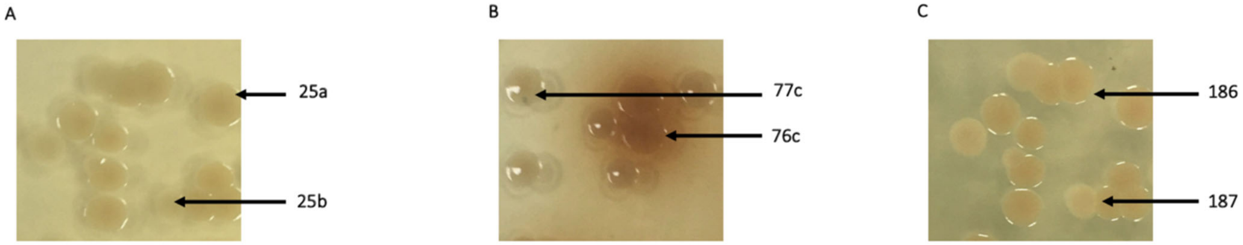

Figure S2. Colony aspects of paired strains on LMB agar at day 2. Morphological differences allowed direct colony counts (A) strains 25a + 25b. Strain 25a exhibited net-edged, dome-shaped, dark beige-colored colonies while strain 25b exhibited net-edged, light beige-colored colonies, (B) strains 76c + 77c. Strain 76c exhibited net-edged, dome-shaped large beige-colored colonies expressing brown pigment, while strain 77c exhibited net edge, dome-shaped large beige-colored colonies without any secreted pigment, (C) strains 186 + 187. Strain 186 exhibited net-edged, dome-shaped, light rosish, colonies while strain 187 exhibited unclear edges, flat, beige-colored colonies.

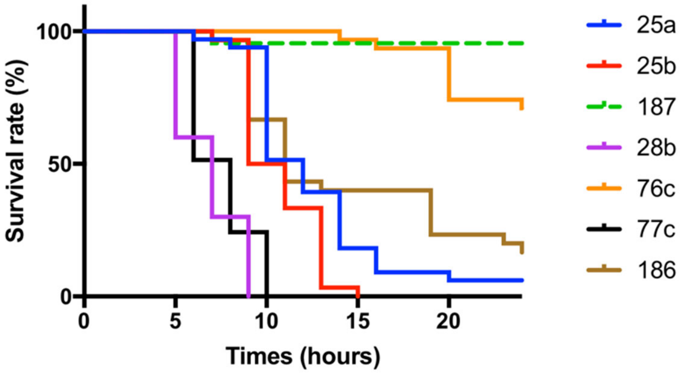

Figure S3. Survival curve of wild-type (WT, w1118) ***Drosophila melanogaster*** **infected by monomicrobial infection.** n=30 fly per condition. Average inoculum: 500 CFU/Fly.
